# Utilizing Blockchain Technology for Healthcare and Biomedical Research: A Review

**DOI:** 10.7759/cureus.72040

**Published:** 2024-10-21

**Authors:** Paras Shah, Chetna Patel, Jaykumar Patel, Akash Shah, Sajal Pandya, Brijesh Sojitra

**Affiliations:** 1 Pharmacology, Government Medical College & New Civil Hospital, Surat, IND

**Keywords:** biomedical research, blockchain, hash, healthcare, patient-centric care, patient data privacy and security, pharmaceutical piracy

## Abstract

Blockchain is a decentralized, secure, and immutable public ledger that offers significant benefits over conventional centralized systems by preventing data breaches and cyber-attacks. It has a great potential to improve data security, privacy, and interoperability in healthcare and biomedical research. This review discusses the basic principles and the historical evolution of blockchain and evaluates the implications of blockchain for the existing healthcare infrastructure. It also highlights blockchain technology’s advantages in electronic health records, supply chain management, clinical trials, and telemedicine. However, this technology faces several hurdles, including regulatory issues, technical complexity, and economic costs, which suggest a gradual adoption over time. In addition, the review emphasizes its ability to ensure data integrity, enhance collaboration, and protect intellectual property in biomedical research. This review shows that blockchain can enhance healthcare data management by providing secure, efficient, and patient-centric solutions. Furthermore, it also discusses the implications of blockchain for the future of healthcare and biomedical research and suggests that ongoing research and interdisciplinary approaches are essential for overcoming current barriers and realizing the full potential of this technology. Future research should focus on developing privacy-preserving hybrid data storage solutions that comply with international laws and regulations, thus enhancing the sustainability and scalability of this technology in healthcare.

## Introduction and background

Blockchain technology has its roots in the year 1982, when cryptographer David Chum first proposed a blockchain-like protocol [1]. In 1991, Haber and Stornetta presented their work on cryptographically secured chains of blocks [2]. Eventually, in 2008, Nakamoto introduced the first decentralized blockchain in his white paper on Bitcoin [3]. This serves as a public ledger for all transactions of the cryptocurrency Bitcoin.

It is important to examine the underlying assumption of the association between blockchain technology and healthcare. Developed nations spend nearly 10% of their GDP on healthcare, and developing nations are continuously increasing their budget allocation to healthcare as expenses for these services are rising, but the scattered patient data is a matter of concern. Blockchain technology promises a transforming future in the healthcare sector by addressing several critical issues and improving various aspects of healthcare management. The core feature of blockchain technology in healthcare is its ability to provide a decentralized and distributed framework that enhances data security, privacy, and interoperability without the need for a third trusted party (TTP) [4,5]. In simpler words, a blockchain is a digitized, decentralized, and distributed public ledger that records transactions in a secure and tamper-proof manner [6,7]. One of the fundamental benefits of this technology in healthcare is the efficient management of patients’ electronic health records (EHRs), which enables data sharing across institutions while ensuring privacy and security [8]. Since no single entity controls the entire data set, this decentralized framework protects against the risk of data breaches and cyberattacks [4]. Additionally, blockchain technology provides robust security measures for data transmission and storage, exceeding conventional methods like Rivest-Shamir-Adleman (RSA) and Diffie-Hellman algorithms [9].

In spite of its potential, the successful development of blockchain for healthcare applications encounters numerous and complex challenges, including regulatory concerns, technical issues, safeguarding privacy, and economic costs [10-12]. These hurdles indicate that the utilization of this technology in healthcare is a gradual process and will evolve eventually [10,13]. Adopting the blockchain in healthcare satisfies data governance challenges by providing a transparent and immutable ledger for data transactions, which can help in complying with regulatory requirements and ensuring data integrity. Blockchain’s decentralized nature solves the “information isolated island” problem in centralized healthcare systems [14]. Moreover, it empowers users by giving them control over their health data [14]. This patient-centric approach ensures that patients possess ownership and control over their data through cryptographic keys and smart contracts that govern data access within the blockchain ecosystem, which results in greater patient satisfaction and engagement [15]. Aiming for the widespread adoption of blockchain in healthcare, the establishment of technical and industry standards by organizations like the Institute of Electrical and Electronics Engineers (IEEE) Standards Association is crucial [16]. These benefits collectively contribute to better healthcare outcomes and more efficient healthcare systems.

This review highlights how blockchain technology can be utilized in the healthcare industry in the future by reviewing its current acceptance and potential.

## Review

Overview of blockchain technology

A comprehensive literature search was conducted using databases such as PubMed, IEEE Xplore, and Google Scholar. Keywords included "blockchain," "healthcare," "biomedical research," "data security," and "interoperability." Articles were selected based on relevance, with a focus on studies, reviews, and case reports published between 2012 to 2024.

**Figure 1:**
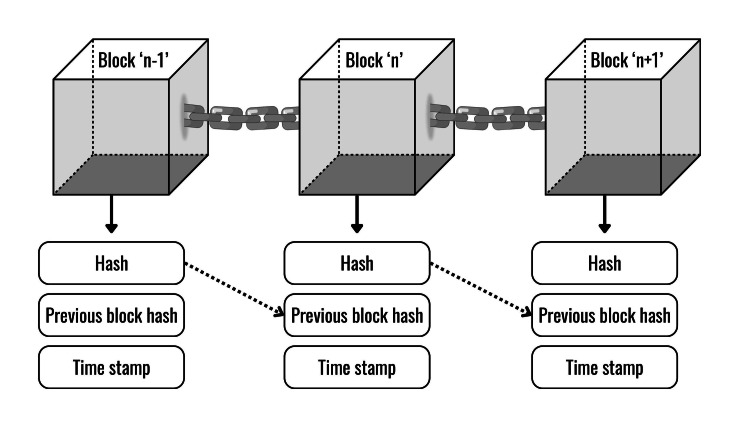
Architecture of Blockchain Image Credits: Paras Shah

One of the fundamental features of blockchain is decentralization. In simple terms, it records transactions across multiple computers (peer-to-peer (P2P)). A node is simply a device running the software of blockchain. These computers are individual nodes, and they are mutually connected. They store all copies of the ledger in a synchronized manner [17]. Each node has the ability to leave and rejoin the network on demand. After leaving the network, it receives the ‘Proof-of-Work (PoW)’. PoW works as a consensus mechanism that provides evidence of the node’s computational expenditure [17]. When certain transactions take place at a node, it stores the data in a block with a timestamp [18]. Each block has a unique alphanumeric code, called a hash (Figure 1). It acts as a digital fingerprint and a link between two consecutive blocks to create a chronological system. PoW determines the order of blocks linked together. This makes the data tamper-proof and immutable [19].

Blockchains can be of three types: (1) public, (2) private, and (3) consortium blockchains [20]. Public blockchains, like Bitcoin and Ethereum, are open to anyone. Private blockchains are restricted to specific participants. Consortium blockchain offers a balance between transparency and privacy as it is governed by a group of organizations.

Applications of blockchain in healthcare

Blockchain technology enhances data security and protects patient’s privacy [21]. To ensure effective patient care, it provides a unified network where different service providers can access patient information seamlessly [6,12,22]. Blockchain offers transparent and traceable records of all transactions and the facility of smart contracts [23]. These are automatically executed contracts without any need for third-party involvement. The clauses are mentioned in the source code. In healthcare, it can streamline administrative tasks such as insurance claims processing, patient consent management, and supply chain logistics, which in turn increases efficiency [24,25]. The specific applications of blockchain in healthcare are described below.

EHRs

The conventional method of recording patients’ data manually in paper-based forms has many drawbacks, and it is partially obsolete now. Many healthcare providers are shifting to EHRs) as it is easy to maintain, trace, and analyze [26,27]. However, the problem of fragmented patient data, lack of interoperability, compromised security, and privacy raises concerns [28]. To address these issues, blockchain technology could be employed [4,11]. The use of public-key infrastructure-based asymmetric encryption and digital signatures provides another layer of security to EHR data [29]. MedRec (Massachusetts Institute of Technology, Cambridge, Massachusetts, United States) is a platform that uses blockchain to provide a comprehensive, reliable, and secure log of medical history. It also allows users to share their data with relevant medical entities across the network.

Internet of Medical Things (IoMT) and Telemedicine

IoMT is a network where multiple medical devices, sensors, and objects are connected with one another. With the help of these entities, remote patient monitoring (RPM) is carried out [30]. Currently, the data collected from these devices are stored, processed, and analyzed locally, which may result in data tampering, single-point failure, and data breaches. This raises security concerns. Integrating blockchain with IoMT creates smart RPM with intact privacy and complete security [31]. BurstIQ (BurstIQ Inc., Colorado, United States), Solulab (SoluLab Inc., California, United States), Medicalchain (Medicalchain SA, London, United Kingdom), Guardtime (Tallinn, Estonia), Avaneer Health (Avaneer Health, Inc., Illinois, United States), Chronicled (Chronicled, Inc., California, United States), ProCredEx (Florida, United States), Robomed (RoboMed Innovations, Bengaluru, India), and Patientory (Patientory Inc., Georgia, United States) are examples of platforms that use blockchain to store, process, and transfer the patient data in a secured manner [17,32].

Network of Body Sensors

Gradually, healthcare is becoming personalized and non-invasive. There are multiple wearables and subcutaneous medical devices available that contain wireless sensors [33]. These sensors form wireless body area networks(WBANs). WBANs are very short-ranged and consume less power as they are designed for communication in, on, and around the human body. WBAN does continuous patient monitoring without hampering the routine activities of a patient. Blockchain safeguards these data transmissions across various platforms [34,35].

Medical Imaging

Currently, medical images are stored and shared on centralized cloud-based platforms. This creates privacy issues. Additionally, it necessitates a vast storage capacity, hefty investments in data centers, and heavy maintenance costs. Blockchain’s integration can make the process more transparent and secure [36]. It only allows the authorized owner to view and share the data with a genuine recipient [37].

Precision Medicine

The aging population will create a demographic shift, worsening the ratio of workers to retirees. Additionally, the increase in chronic diseases further strains healthcare systems with age-related diseases, such as Alzheimer’s disease (AD). AD has no cure, and hence early diagnosis and precise treatment are very important to improve disease outcomes [38]. In the case of type-1 diabetes mellitus (T1DM), the transition from pediatric to adult care is a high-risk period. A personalized transition program is essential for better health outcomes [39]. Robotic microsurgeries are becoming popular, and they offer benefits like improved precision and reduced tremors in nerve surgery [40]. Overall, multi-system changes and moving beyond just acute care focus are very important steps to adopt precision medicine [41]. Blockchain technology has the potential to provide security and privacy for this upcoming era of personalized medicine [42].

Managing Genomic Data

The future of healthcare belongs to personalized or precision medicine. There are plenty of studies being carried out in this domain, and the expansion of targeted therapies will create a host of challenges [43]. Blockchain can facilitate the secure exchange of genomic data, promoting collaboration among researchers while protecting patient privacy. EncrypGen (New York, United States) and Nebula Genomics (California, United States) are offering blockchain-based services to store, share, and trade genetic information securely.

Clinical Trials and Pharmaceutical Industry

A clinical trial is a long-term journey involving multiple complex processes like target identification, drug discovery, drug development, regulatory approvals, and post-marketing surveillance. It requires accurate data collection and management in order to yield effective outcomes [44]. Tracking patient enrolment across multiple centers, monitoring adverse drug reactions (ADRs), ensuring compliance with regulatory requirements, assessing quality control, and maintaining the supply chain are some of the most crucial steps in any clinical trial [45]. Incorporating blockchain technology in these processes smoothens the entire exercise of clinical trials with features like distributed ledger, PoW, a consensus mechanism, and smart contracts, the programs that automatically execute when certain conditions are met. Blockchain 2.0 and Blockchain 3.0 can even further help to make the process more efficient than ever before.

Pharmaceutical piracy is a major public health concern across the globe. It is defined as the sale of counterfeit and illicit medical products. This occurs when pharmaceutical companies and regulatory authorities lack control over the supply chain of pharmaceuticals and an illegitimate supply chain gets established. This poses a big challenge to the healthcare authorities. Blockchain has the potential to mitigate the risk of pharmaceutical piracy. Wide acceptance of blockchain can eliminate such illegal practices by securing supply chain logistics. Platforms like Embleema (New York, United States), BlockPharma (Paris, France), Tierion (California, United States), and FarmaTrust (Blockchain AI Solutions Ltd, London, United Kingdom) offer blockchain-powered services to accelerate the drug development process and validate the authenticity of pharmaceuticals.

Administrative Processes

A patient’s experience encompasses a series of events, starting from the very first contact with a doctor to the follow-up services given by the hospital. These events demand a lot of effort and time. Blockchain simplifies administrative processes, including insurance claim processing, to a great extent with the help of its fundamental features like smart contracts, interoperability, decentralization, and a unified network that boosts administrative procedures [46,47]. Coral Healthcare (Coral Healthcare Pvt. Ltd, Kolkata, India) uses blockchain to streamline various administrative processes.

Thus, blockchain can provide a sustainable solution for healthcare data management and help in creating a patient-centric digital healthcare framework [48,49]. It can increase the rate of correct evidence collection and minimize authentication issues, thereby enhancing the scalability and sustainability of the healthcare system [50]. A proof-of-concept conducted with a medical clinic demonstrated the feasibility of integrating blockchain into healthcare systems [47].

Blockchain in biomedical research

Over the period of time, biomedical research has undergone many administrative and fundamental changes. Especially after technological advancements like high-speed broadband connectivity, cloud computing, remote data processing and data storage, artificial intelligence (AI), machine learning (ML), natural language processing (NLP), and the development of large language models have driven a paradigm shift in this shift. With the help of these tools, researchers are moving towards ‘multi-centric studies’ on a large scale [51,52]. Genomic data is also being utilized in this research. Multi-centric studies mean data exchange and data sharing among different entities to integrate multivariate data for the development of patient-centric therapies [53]. Achieving seamless integration and communication between different systems remains a significant challenge [54].

However, dealing with large data sets brings a few ethical challenges, like the protection of an individual’s right to data privacy [53]. Since this data is highly sensitive, it is extremely important to ensure the security of this data [55,56]. Generally, big institutions that are conducting biomedical research predefine the roles of different stakeholders. Subjects provide personal data, data collectors collect that data, and data utilizers or controllers define the purpose of data use. Hence, there’s a significant transparency issue between subjects and data collectors as well as utilizers, which deprives subjects of control over their data, leading to possible data harm [57].

Since blockchain has fundamental features like decentralization, immutability, traceability, data security, data privacy, interoperability, efficient data management, and transparency, it has emerged as a transformative tool in biomedical research [58]. The primary advantage of blockchain in this domain is its potential to enhance data security and integrity. It can help in eliminating data falsification and underreporting in clinical trials, ensuring that all results, including undesirable ones, are accurately reported [59]. Blockchain enables subjects to control their data and prevents data harm. Moreover, it provides a transparent peer review process to publish scientific work [58].

Challenges

Lack of Comprehensive Evaluation

Despite the caliber of blockchain, its use in the biomedical domain has not been properly investigated for implementation and evaluation. This gap in research limits the understanding of blockchain’s practical benefits [60].

Barriers to Real-World Adoption

Existing prototypes of blockchain-based healthcare systems encounter significant legal, social, and technological limitations, which act as barriers to adoption [61,62].

Limited Real-World Implementations

Most blockchain implementations are in the infantile stage, with very few being deployed in healthcare facilities. This limited real-world application restricts the ability to fully understand and address the practical challenges of blockchain in healthcare [60].

Inconsistencies in Metrics

Studies suggest that there are inconsistencies in the metrics, particularly regarding storage and query speed. This makes it difficult to compare different blockchain technologies and assess their feasibility for data management [60].

Interoperability Challenges

Budding technologies, protocols, standards, and frameworks in the healthcare ecosystem demand seamless integration and communication among different systems [54].

Need for Interdisciplinary Approaches

To establish patient-centric therapies and to provide innovative solutions for managing medical data, the focus should be on interdisciplinary approaches rather than monodisciplinary approaches [63].

Implementation Cost

Blockchain consumes significant computational power, and some of these platforms utilize Ethereum technology, which is costly [64].

Data Storage Limitations

Stakeholders are concerned about the amount of data that can be stored in the blockchain. Furthermore, blockchain gives permanency to data, which may violate Europe’s General Data Protection Regulation’s (GDPR) Article 17 - Right to Erasure, which grants citizens the right to request the modification and deletion of personal data [65,66].

Therefore, addressing these challenges is necessary to realize the full potential of blockchain technology.

Future directions

Future research directions include designing privacy-preserving hybrid data storage and interoperable infrastructures that comply with international laws and regulations [61]. Furthermore, to extend our current knowledge, future studies should focus on developing standard protocols for blockchain implementation. Possibilities of blockchain integration with other new-age technologies like AI, ML, NLP, and extended reality (XR) should be explored for practical benefits in healthcare delivery systems. [67] Additionally, to establish interoperability across different blockchain-based platforms, open standards must be developed to enhance compatibility.

## Conclusions

Our review highlights that blockchain technology offers solutions for data security, privacy, and interoperability. Blockchain’s decentralized nature significantly reduces the risk of data breaches, cyberattacks, and frauds. However, its immutability ensures data integrity. Blockchain’s potential to provide transparent and traceable records is extremely important for maintaining the authenticity of healthcare data. This leads to increased patient compliance, trust, engagement, and compliance. Moreover, it empowers patients by giving them control over their personal health records, fostering a patient-centric approach. In practice, these findings can be applied to enhance data management and security. Blockchain can be deployed to create secure, interoperable platforms for EHRs, enabling seamless data exchange between healthcare providers and different stakeholders in biomedical research. Additionally, blockchain's traceability features can optimize supply chain management for pharmaceuticals, ensuring the authenticity and safety of medications. Albeit, several concerns like regulatory challenges, technical complexities, and high implementation costs need to be addressed to realize blockchain’s full potential in healthcare. Integrating blockchain with existing healthcare systems is a crucial step to ensure data safety and achieve interoperability.

To deepen our current understanding, future studies should focus on developing standardized protocols for blockchain implementation in healthcare. Its integration with other emerging technologies should be explored to assess the practical benefits. These studies should also address ethical challenges like patient consent and data ownership. Thus, blockchain technology can revolutionize healthcare and biomedical research by evaluating new opportunities, which will lead to more secure, efficient, and patient-centric systems.
